# Entering an emotional minefield: professionals’ experiences with facilitators to address abuse in child interviews

**DOI:** 10.1186/s12913-019-4128-8

**Published:** 2019-05-10

**Authors:** Ane Ugland Albaek, Per-Einar Binder, Anne Marita Milde

**Affiliations:** 1 The Department of Clinical Psychology, University of Bergen, P.O Box 7807, N-5020 Bergen, Norway; 2 The Department of Psychosocial Health, University of Agder, P.O. Box 422, N-4604 Kristiansand, Norway; 3 The Regional Centre for Child and Youth Mental Health and Child Welfare, NORCE Health, Bergen, P.O. Box 7810, N-5020 Bergen, Norway; 4 The Department of Biological and Medical Psychology, University of Bergen, P.O. Box 7807, N-5020 Bergen, Norway

**Keywords:** Exploration, Child abuse, Health care professionals, Social workers, Facilitators

## Abstract

**Background:**

Extensive research documents that child abuse is widespread and that it has detrimental effects on victims’ physical, psychological and social well-being. Efforts to help abused children by removing stressors and administering restorative care can reverse these negative effects, but the evidence suggests that professionals often fail to expose child abuse. This study aims to generate insight into professionals’ experiences with facilitators in handling the challenges of addressing abuse in child interviews. We expect that this knowledge can improve interventions that qualify professionals in the identification, protection and care of abused children.

**Methods:**

Within the qualitative approach and an Interpretive Description framework, we performed in-depth interviews with nineteen participants from southern Norway, specifically ten social workers from child protective services and nine psychologists from child mental health services. Then, Interpretive Description analysis was performed by using constant comparison, reflexive and critical examinations, and contextualized theoretical interpretations.

**Results:**

The participants’ accounts revealed that various facilitators relative to the stages of the skill development and intrinsic motivation of the practitioner enhance the explorative work of the professional. We identified the following five main themes: (a) *alleviating personal choice*; (b) *collective accountability*; (c) *sharing vulnerability*; (d) *finding your own way*; and (e) *doing it for the right reasons*.

**Conclusions:**

To facilitate explorative work, our findings suggest that competence development should apply goal-directed reflective practice combined with positive feedback on performance. Furthermore, our results indicate that developing personal competence is contingent on supporting individual choice and volition while decreasing demands towards following rules and guidelines. To promote the relatedness and the emotion regulation of professionals, we suggest endorsing shared vulnerability with colleagues and promoting an organizational culture that supports openness and allows professionals to discuss their emotions when addressing difficult and complex issues. It is also advisable to promote autonomy by helping professionals to find meaning in their work that is compatible with their personal values.

**Electronic supplementary material:**

The online version of this article (10.1186/s12913-019-4128-8) contains supplementary material, which is available to authorized users.

## Background

Reports indicate that professionals often feel that they lack the necessary resources to explore child abuse when conducting child interviews. Professionals are also afraid that they may make matters worse for the child, given that exploring abuse induces such strong negative emotions [1, 2]. Akin to walking a child into a minefield, professionals fear the unpredictability and potential harmful effects to both themselves and the child when broaching the subject of child abuse [1]. Although traditional training and education include theoretical knowledge of child abuse, the methods and assessment tools for addressing child maltreatment, action guidelines, and standardized procedures seem to be unable to resolve the complex challenges with which professionals struggle when interviewing children [1, 2]. Moreover, evidence from studies indicates that professionals in child protective services (CPS) and child and adolescent mental health services (CAMHS) are often unsuccessful in uncovering children’s adversities [3–5].

Numerous studies from several disciplines document the detrimental and long-term effects of child abuse, including psychological, physical and sexual abuse and neglect [6–9]. Moreover, maltreatment can impede a child’s neurobiological development and cause cognitive, emotional, and relational deficiencies followed by an increased probability for physical and psychological disease, disability, and mortality in adulthood [10]. Studies also indicate a high frequency of child abuse in both Europe and the US [11–13]. Therefore, we must ensure that children are protected from abuse, and we must swiftly offer restorative care to children exposed to such abuse to minimize both the short- and long-term negative consequences. Nevertheless, few abused children who exhibit symptoms that warrant clinical attention receive effective treatment [14, 15]. Therefore, we must assess children’s adversities correctly and systematically to diminish further exposure to stressors and reduce their harmful effects. Consequently, the inadequate exploration of abuse within child services may be a key issue that prevents efficient assistance to child victims [3, 16–18]. For instance, research reveals that many victims of child abuse do not disclose their experiences during childhood [5], despite their contact with CPS or CAMHS professionals [3, 18]. Piltz and Wachtel’s [19] review of quantitative research reveals that nurses’ suspicions of child abuse depended on the individual practitioner’s personal characteristics, such as their knowledge, experience, fear of perceived consequences, and lack of emotional support. In a meta-synthesis of qualitative research [1], professionals struggled to address abuse in child interviews because of their emotional discomfort, the complexity and unpredictability of broaching the subject, and the belief that they lack sufficient knowledge and skills to address the issue. Research regarding the origins of professionals’ emotional strain when exploring child abuse emphasizes their perceived lack of resources and ability to effectively aid abused children and their concern about their personal empathic involvement [2]. The lack of empirical studies on how professionals overcome their challenges when addressing maltreatment during child interviews indicates a substantial knowledge gap. Accordingly, the aim of the present study is to address the following research question: based on the experiences of professionals, what are the facilitators that allow them to handle the challenges associated with exploring abuse in child interviews?

## Method

We used Interpretive Description (ID) methodology [20, 21] to investigate the clinical phenomenon of addressing child abuse while also ontologically and epistemologically applying hermeneutic phenomenological methodology. ID methodology is an applied qualitative approach to address complex experiential questions in health disciplines and uncover subjective and experiential knowledge that can inform clinical practice. Heidegger’s [22] phenomenology inspired our approach of the contextual investigation of the participants’ lived experiences aimed towards interactively constructing meaning patterns. Heidegger advocated that a person’s lived experience is an interpretive process that occurs intersubjectively through communicative signs and language. Our goal to understand the participants’ lived experiences with facilitators when addressing child abuse led us to be inspired by Heidegger’s concept of the hermeneutic circle, whereas research entails a continuous movement between questions and answers and between implicit preunderstandings and explicit understandings. Similarly, Gadamer’s [23] dialogical hermeneutics, which emphasized how all interpretation is a result of the fusion between the horizons of the interpreter and the interpret, also influenced our research. Moreover, Gadamer argued that we must explore a phenomenon from different angles to understand its various aspects. Consequently, when researching the participants’ experiences with aspects that facilitated explorative work, we tailored our interviews and our analysis; thus, we could view the phenomenon from different angles. Consistent with both Heidegger’s phenomenology and Gadamer’s hermeneutics, we have an epistemological perception of research such that it is a product of the complex interplay among the informants, the research process, the context, and the actions of the researchers [24, 25]. Because of this perception, we practiced reflexivity during all parts of the project to identify our preconceptions and how they influenced our interpretations [26, 27]. ID methodology contributed to the achievement of our study goal because the methodology is constructed to identify themes and patterns from participants’ subjective perceptions while acknowledging the researchers’ clinical foreknowledge and expertise as influential and beneficial to the research process [20]. We collected data through informed questioning, reflexive critical examination, and contextualized interpretations consistent with ID methodology [21]. Most importantly, ID methodology seeks to generate findings not only in the form of isolated themes but findings that form a coherent professional narrative that experts in the field perceive to be convincing. Thus, the research product should expand clinical understanding and propose practical applications [21].

### Recruitment

The first author telephoned seven leaders of CPSs and CAMHSs to introduce the project and ask for permission to interview their employees. All leaders accepted and were subsequently sent an e-mail that delineated the study. Some leaders selected participants and arranged interviews, other leaders forwarded the e-mail and invited interested employees to contact the first author, and one leader shared the employees’ contact information with us and allowed us to make direct contact with the employees. We have no knowledge of any employees who declined participation.

### Participants

We interviewed 19 participants, namely, 10 social workers (two males) from CPS and nine clinical psychologists (one male) from CAMHS. Because our aim was to investigate professionals’ experiences with facilitating aspects to address abuse in child interviews, participants with specialized competence and work experience in the field of child abuse were included. The Norwegian CPS is responsible for upholding children’s right to protection from abuse and neglect. They uphold this right by counselling parents, investigating suspected maltreatment, presenting legal claims for child removal, reporting suspected parental legal violations to the police, and providing alternative care. When someone is concerned that a child may be experiencing abuse, they usually report it to CPS. The CPS participants in this study were assigned to investigate suspicions of child abuse and worked in three separate CPS offices that varied in size, geographic location, organization, and demographic area (urban, suburban and rural). The inclusion criteria were a degree in social work and work experience that involved suspected child abuse cases. The participants’ work experience ranged from one to 35 years (median 14), and they worked with different client age groups, i.e., preschool and elementary school children and adolescents. The Norwegian CAMHS provides services to children with mental health challenges through individual, group, and family therapy, which means that CAMHS workers meet with many troubled and potentially traumatized children. The inclusion criteria for the CAMHS workers were a degree in clinical child psychology and clinical experience with children exposed to abuse. The CAMHS participants worked in four CAMHS offices that varied in size, geographical location, and organization. These participants had between 10 and 35 years of work experience (median 20), and all CAHMS participants supervised colleagues with lower levels of education.

### Data collection method

The first author conducted singular, semistructured, in-depth interviews with all participants. As geographic dispersion created a travel distance, we chose to interview the participants at their workplace to increase the response rate. We developed an interview guide to ensure that key areas of interest were covered, and we supplemented these questions with exploratory spontaneous questions to maximize the collected data [28]. Our interview guide included the following: the participants’ successful and challenging experiences with respect to abuse exploration; their thoughts regarding the impact of individual differences; their personal experiences, work experiences and relationships; their perceptions of facilitators, barriers, and improvement strategies; and their suggestions for interventions to improve abuse exploration (see the translated interview guide as an Additional file 1). The mean duration of the interviews was 68 min, the range was 44 to 97 min and the median was 74 min. We transcribed four interviews verbatim, and a professional firm transcribed the remaining interviews by using the same transcription template. All coding and further analyses of the transcripts were performed by the authors. We checked all transcripts for inaccuracies. The 19 transcribed interviews constitute the data for this study.

### Researchers

The first author is an organizational psychologist experienced in competence development for professionals and leaders within health and social services. During the data collection, she worked at a regional center for psychological trauma. The second author is a professor in clinical psychology who studies psychotherapy and change processes and is also an experienced psychotherapist. The third author is an experienced clinical therapist and an associate professor in biological psychology who conducts experimental and clinical research on stress and psychological trauma. Our motivation for this study was based on a joint desire to help abused children. However, we were also influenced by research that indicates that many abused children remain undetected, despite contact with various agencies and aid services. Based on our professional experiences as clinicians and as educators of professionals, we all had a presumptive understanding that addressing child abuse is difficult. Moreover, we also believed that the challenges associated with exploring child abuse are not well-understood and are not often discussed.

### Data analysis

Adhering to the general principles of ID methodology, our analytical progression was inductive and entailed constant comparative analysis to extract commonalities and discrepancies between and among the participants with respect to the research question. ID methodology favors coding with a focus on a broad overall picture rather than a line-by-line focus, which is advocated in content analysis. Therefore, we identified codes through reading and constant comparisons and assigned broad and descriptive titles to our emerging codes to capitalize on their potential and expand the analysis with the assistance of Nvivo 8 software [21]. Next, we identified initial meaning units endorsed by intriguing quotations. Evolving in our analytic process, we immersed ourselves in the initial meaning units and explored the data set as a whole to develop and expand our thematic insights. Finally, we searched for conceptual relationships and explored remarkable and divergent meaning units and quotes in relation to the research question. We chose to work as a team with critical reflexive discussions that were enhanced and contested by our diverse experiential and professional backgrounds. In several stages, we described proposed themes with representative quotes, discussed these themes, and then replaced them with improved themes until we developed an organizational structure that we agreed conceptualized the most meaningful set of findings [20].

## Results

Our analysis of the participants’ accounts of the aspects that facilitated their management of the emotional strain associated with addressing abuse in child interviews produced the following five main themes: (1) *alleviating personal choice* describes how the participants perceived routines and tools to facilitate exploration and alleviate their individual responsibility; (2) *collective accountability* delineates how the participants allayed their doubts through consultations with other professionals; (3) *sharing vulnerability* incorporates openness and emotional support as ways to assist the participants in coping with the challenges associated with addressing abuse; (4) *finding your own way* involves the participants’ agreement that overcoming the challenges of explorative work requires courage, reflective experience and practice; and (5) *doing it for the right reasons* means that the participants found it easier to endure stress when they believed that they could make a difference in the lives of abused children. All of these themes were expressed by both the CPS and CAMHS participants with only minor intergroup variances. In the following presentation, “all” participants equals all 19, “most” is equal to 12 to 18 participants, “some” means 5 to 12 participants, and “a few” refers to less than 5 participants.

### Alleviating personal choice

Most participants found routines, guidelines and assessment tools to be beneficial in initiating abuse exploration during child interviews. Routines supported them in overcoming inner resistance to asking children about abuse, and they also alleviated their personal responsibilities for making decisions. On the downside, some participants thought that routines and forms were inefficient and served ulterior motives.

Most participants reported that asking about maltreatment caused them to feel mean, and they then feared that they would lose the children’s trust. However, an obligation to routinely explore abuse helped many participants to overcome these obstacles, as they could then legitimize their inquiry of the child.

…But I try to sugar-coat it a bit, that it’s something I ask everybody. We’ve decided that we have to ask everybody, so that’s what I do. When I put it like that, it’s less uncomfortable to ask. [...] I think it’s helpful to use a form as a starting point (#10).

To tell children that they asked everyone these questions made it easier for the participants to raise the subject. Some participants emphasized that explicit routines provided safety for both the professionals and the children. “Even though there’ll always be things that’ll be difficult, the clearer the guidelines and routines, the more secure the framework will be for us, and it’ll be safer for the child” (#3). The participants’ understanding of children’s safety referred to fair, high-quality services that allowed children to disclose abuse. Some participants also stated that by telling children that the reason they asked everyone is because abuse is common, the children would feel more normal. Similarly, the safety of the participants involved relief from solely depending on their own interpretations and suspicions. One participant explained, “Previously, I acted on my gut feeling that there was something like that through observing the interplay – my impression that this child is hiding something. But now we must ask about it anyhow” (#17).

When the participants had direct orders to always ask about abuse, they found it easier to ask about abuse. “Of course, when it comes to interviews with children, and it’s written that we must investigate, you go more into it than if it didn’t say that it was mandatory” (#19). Another advantage to routinely addressing maltreatment was that it became easier to remember to address maltreatment. “The explicit procedure saying that we always have to ask about psychological trauma creates a benchmark for us. It makes it easier. Then, it’s like we can tick the box – we’ve done it. It turns into a kind of checklist” (#14).

Because most participants found it difficult to determine how and when to ask about abuse, some believed that routines and assessment tools helped. “What makes it easier is that there are clear procedures or guidelines on *how* we should do it and maybe *when* we should do it. [...] I’m very fond of using those forms, and I think it’s great that we have them” (#14). Some participants said that they followed routines without thinking about adhering to them. These participants regarded this as testimony to the routines’ efficiency. “There’s a lot we have routines for where I don’t think about it being routine. And I believe that’s a good routine because you don’t think, ‘Oh right, I must follow that routine,’ you just do it” (#6).

Even if most participants said that they used routines and assessment tools, they agreed that these played a miniscule part in the disclosures by children. One participant laughingly summed up the limitations of the assessment tools. “The dream would be to have a tool that told us exactly what questions to ask to get a correct answer” (#6). Another claimed that routinely asking everyone about abuse during a first or second session only elicited disclosure from the children who had already decided to tell. “Those [children] who aren’t ready or can’t do it don’t tell right away no matter what. A superficial investigation is not enough to achieve disclosure” (#16).

Some participants were critical of the extensive use of routines and forms because they believed that some routines were used to limit liability or to document task completion rather than promoting and ensuring the best interest of the children. In fact, some participants perceived that routines could be obstructive.

Routines are important, but I have a pet peeve. I believe in routines, but sometimes, I think you have them just to look good. You shouldn’t create a routine just because it’s a routine but because it’s something that works. Having a routine that doesn’t work is much worse than having no routines. And I’ve experienced that here, we have a routine just because there must be a routine (#6).

Many participants were afraid that their appraisals and decisions would be challenged by outside parties, and they used guidelines to safeguard themselves from critique. “If we have doubts about something, then we have to check, and then, we’ve covered our backs because at least we’ve checked the guidelines” (#8). Thus, guidelines could induce a false sense of accomplishment for adhering to them while suspending professional judgement.

### Collective accountability

Most participants expressed that they felt more confident after discussing difficult calls with colleagues, counselors and leaders. When they were unsure of how to interpret children’s responses and how to act, these discussions allowed them to share accountability for their interpretations and decisions with other people, which therefore eased their fear of critique or of making errors. Additionally, asking other people for their opinions made them feel more confident in the quality of their work.

If it’s been discussed in the group and with the second caseworker, it’s not just my point of view that led to this conclusion. Then, if there’s still doubt, we have a forum we can confer with to get a broader deliberation. That makes it easier. Court cases are even less stressful since *we* don’t make the final decision (#7).

Many participants thought that routine group discussions functioned as a quality assurance measure.

All of our cases are discussed after 3 months or 6 months. We check if a diagnosis is set or if we need help. It’s a system to ensure all cases are regularly discussed by a multidisciplinary team. And that’s a [...] quality assurance for us. Because if you’ve forgotten to ask about it, then at least someone will mention it at the evaluation (#14).

This routine made the participants collectively accountable for the case work, including any errors committed. Correspondingly, having their leaders review their work reassured some participants. “I think it’s important that someone does a quality check of important documents and appraisals that we make. It shouldn’t be *one* person making all the decisions that we do. We need supervision. It’s imperative that someone oversees us” (#19). Many participants emphasized the importance of having a colleague with whom to collaborate when appraising a child interview and deciding what to do. One participant stated that “To reassure the workers that they’re not alone [...] because being the only one thinking about it can be a lot. [...] We need someone to turn to and not be left alone in appraising these cases” (#8).

A few participants with short work experience felt reassured when working with experienced colleagues.

If you’re working with someone with extensive work experience, then you have such reassurance [...] to confirm that the approach is good and that we’ll do it this way. It creates lots of security right away, and you get a sense of confidence in what you do. [...] I’m on a team that feels good to be on, especially for me as a newcomer, because I have many experienced social workers around me (#3).

In organizational cultures that emphasized abuse-disclosure, many participants found exploration easier. “I think in our culture, everybody agrees that you should ask about it. And that probably makes you more alert to it” (#10). Likewise, many participants highlighted the importance of their leaders’ explicit instructions to address abuse. “What’s so reassuring is that we know that our leaders’ attitudes concur with our own, and then it becomes so much easier to work with it [...] The leaders are very, very important” (#4).

Interestingly, one participant shared that when her heavy workload reduced the quality of her work, she handled her frustration by allocating the responsibility for this to her leader. “I’ve become more outspoken over the years, like ‘okay, I’ll take that family too if my leader decides that, but then you have to know that the quality will be so and so.’ It does weaken the quality. But if everyone acknowledges that and finds it acceptable, then it’s fine by me” (#5). Therefore, attributing the reduced quality of her work to reasons beyond her control allowed her to maintain her self-efficacy.

### Sharing vulnerability

All participants described their work with potentially abused children to be so challenging that they needed emotional support to cope. The participants valued comfort from colleagues, a culture for sharing vulnerability in the workplace, and recognition of their worth as a professional.

Most participants emphasized the importance of comfort from colleagues when they felt overwhelmed by difficult emotions. They explained their needs in different ways. “To get counselling to work on myself so I can feel secure that I can handle the information I get” (#17). “Of course, it’s vital to be able to talk to colleagues if you’ve heard things that are difficult to relate to” (#10). Trusting that their colleagues would take time to understand and comfort them made the emotional turmoil more tolerable. As one participant stated, “To be allowed to *disturb* others, your colleagues, because they know that it’s not always easy” (#19).

The participants agreed that an open, supportive culture was crucial for efficient collaboration. “Working in a team requires a great deal of security and openness… you know each other and dare to say things, because it’s mostly about that” (#9). A key element to this openness was admitting personal vulnerability, shortcomings and insecurities.

That I can say things such as ‘You know what, I find this uncomfortable’ and be certain that they won’t think that I’m dense or ‘you should get it together.’ It’s never anything like that. And we can talk about everything (#6).

“To have good colleagues…and places where you can discuss things and dare to ask stupid questions” (#5) was identified as vital for collaboration. For example, expressing doubt about their own ability to cope could have the paradoxical effect of increased mastery. “Recognizing your own insecurity is also a qualification, and then, you have places to go and people to ask” (#13). Although many participants had colleagues to whom they could turn, they actually solicited more the collective sharing of vulnerability and emotional reactions. “To have an open dialogue on how [discussions of abuse] can affect you psychologically and what it does to you to have these interviews, I’m sure that would have helped” (#1).

The participants’ own self-doubt and emotional distress resulted in insecurities about their worth as professionals. Many shared a wish for other people to recognize their personal value and competence, such as “Not just the case work but also security, personal security that I’m doing a good enough job” (#14). “[You want] To receive some support that the way you acted was good and that it’s tough to listen to, and for someone to support my decisions [...] We do need help from the ones we’re working with to have faith in ourselves, at least I need that. I need someone to give me a ‘thumbs up’ now and again” (#19). “To become self-assured, you need feedback and someone that can see that you do good things, too” (#5). When asked what advice she would give other therapists, one participant said “You *know* this. Us devoted therapists have lots of competence and there are no magic tricks or exact solutions, rather it’s about using your general competence combined with openness and access to support from colleagues” (#13). One participant explained how feeling significant promoted coping.

All these things are very important. It’s about yourself feeling supported, feeling seen and through that, maybe feeling significant? This makes you want to go to work even if it’s a difficult job. “So, it’s important because I know from experience that if you start to struggle and become more and more invisible and no one catches you, it’s not long before you’re in that spiral, and it becomes too hard to do your job” (#7).

Thus, handling difficult emotions became easier when the participants shared their endeavors and their situations with their colleagues. Although most participants expressed gratitude for their colleagues’ emotional support and recognition, many wished for greater shared vulnerability and openness about difficult emotional reactions.

### Finding your own way: practice, practice, practice

All participants agreed that handling the challenges of exploring abuse depended on reflective experience and practice. They each had to develop their own way of performing their professional role through bravery, experience, practice beforehand, episodes of mastery, and an analysis of their performances and feedback.

Because addressing abuse evoked strong emotions, most participants believed that it required courage and willpower. “It’s about daring to ask and not be afraid of missing the mark or making a fool of yourself” (#16). “You must dare to be honest and direct. Dare to ask the difficult questions” (#18). Some participants found that risking to explore abuse taught them that the discomfort was manageable. “I don’t know if it’ll feel less stressful, but what I’ll say is, ‘Dare to talk about it.’ What I’ve learned over time is that it’s not so dangerous to talk about it, to dare to put into words what it’s really about” (#7).

Most participants found that practicing beforehand reduced their discomfort and insecurities. Some talked about practicing with colleagues. “To practice your interviewing technique is key. It’s important to become comfortable interviewing and keeping a person on track. It varies with the degree of taboo linked to the questions, but I think it’s about training, experience, and being prepared” (#17). “If I think something might be uncomfortable, I do it anyway, but first I talk to someone about it. I get counseling, role play, or reflect on it. [...] To practice talking about it with each other is how you learn that it isn’t that scary after all” (#4). One participant recommended practicing alone. “Use the mirror, find sentences that are yours and repeat them. Rehearse your tone of voice. Notice how you may signal that now I’m asking something that I don’t really want an answer to, notice your gaze, your voice… Get to know yourself” (#17).

Experiences of mastery increased most participants’ level of confidence. “When you have a fair share of experiences of mastery where you contributed…the more of these positive experiences you have, the more self-assured you become that I *can* do this” (#19). “It’s about my experience when I have these interviews often and I feel they went okay, and I get feedback from the child that it was fine, or I reveal things and move forward. It strengthens me…” (#8). Some also said confirmation that their past actions were warranted made the tough decisions easier. “Then, at least we’re reassured afterwards that we had the right gut-feeling and responded correctly [...] And then you become more confident in your own decisions [...] You gain confidence that makes it easier to take children seriously next time”. (#1).

Many participants said that they worked hard to improve by analyzing themselves, their practice, and the feedback from other people. “You need to make an effort to perform well and find out what works and what doesn’t. You must invest in it to become good at it” (#6). For some participants, regretful experiences instigated scrutiny from which they learned and then modified their approach. “It’s one of those situations I’ve analyzed… How you meet a child and convey, ‘[it’s] great that you can tell, but you should tell it to someone else.’ This was because I didn’t think. It was done with the best intentions, but I was supposed to be able to take it” (#5). Many participants said that they needed to work on themselves to improve their approach: “To go in depth because we must work extensively on challenging ourselves to learn […] because we must rehearse it to be able to do it” (#19). They realized that becoming aware of their shortcomings was difficult but necessary. “Then, there’s the question of where my blind spots are, and those are more difficult to become aware of. The challenge is the immediate things, the ones I don’t see” (#13). “This disbelief that I have, that’s important to have an ordered relationship to, because it can get in the way of seeing” (#12).

Most participants believed that accumulated experiences facilitated the exploration of abuse in children. “So I think you need volume training to gain the self-assurance necessary for the child to feel it’s okay to tell” (#1). “I think it works pretty well most of the time, but maybe that’s because I’ve done it so many times and feel more self-assured talking about it and to stand to hear what they say” (#2). “For me, it took a few years before everything was comfortable [...], or it can [still] be uncomfortable, but at least you feel that you can take it” (#14).

Training, reflection and experience increased most participants’ understanding of their strengths and weaknesses and helped them to develop a personal work style. “Some need a long time while others learn fast, but it’s about finding your own [way] and being assured in your own role and not conveying to children that you’re insecure and uncomfortable” (#13). “I think it becomes easier and easier the longer I’ve worked. I’m feeling secure enough to create my own standard sentences” (#14). Another participant emphasized that quality depends on authenticity. “Be yourself. Don’t try to be something you’re not. Follow the child in the interview, be present and show that you care” (#7). Some participants explained that being genuine was scary and felt unprofessional although it was crucial for effective interactions. “Maybe just daring to use yourself more. That it’s okay to do so” (#8). Some participants explained that they had developed a certain basic knowledge that guided their practice. “I always ask children, ‘What’s the worst you have experienced in your life,’ and then I assess how the child looks when [he/she] tells me” (#17). Developing a personal style when exploring abuse requires courage and diligent effort through practice, experience and self-reflection. However, when the participants were successful, finding their own ways to address abuse improved their confidence, emotion regulation, and self-assessed performance.

### Doing it for the right reasons – values and intentions matter

All participants described their work helping abused children as meaningful, and many stated that believing that they could make a difference helped them to tolerate and cope with job-related stress. Moreover, for most participants, the conviction that they were doing it for the right reasons mitigated their fears and insecurities, and acting according to their morals and values created and strengthened their self-respect.

To believe their efforts could create positive changes for children was important to most participants: “To believe that it can change. [...] and to later on learn about subsequent improvements in the home makes the work worthwhile. There are some success stories” (#7). “It’s rewarding to work with, and at the same time challenging. It’s worthwhile […] I have very good examples that demonstrate it’s important work and that you can make a difference” (#13). Some participants also said that the ability to do a good job depended on each professional believing that they could make a difference. “You must have a desire to help and believe that your help works […] You must want to change things and have faith that it can happen” (#6). A few participants said that their knowledge of the harmful effects of abuse increased their motivation to help and strengthened their faith in the meaningfulness of their work. “Understanding how extensive it can be to live with violence and abuse...that it affects basic functioning and that it’s beyond just a ‘difficult experience’” (#16). Other participants, however, found these challenges to be intriguing. “At the same time, it’s an extremely exciting job, too. So, it’s full of contrasts really” (#7).

Many participants found it easier to endure the associated discomfort because of their commitment to improving the lives of the children. One participant explained that “When you have a plan in those cases where they’ve begun to talk about violence, I think it’s okay to talk about it. As long as we’re moving in the right direction” (#5). Feeling convinced of the child’s need for intervention and support also made it easier to address abuse during the interview. “It’s easier to ask when the violence is known, because then, it’s on the table, and everybody knows” (#9). Sometimes, the participants shared with the child their reasons for asking about abuse. “I notice that it helps a little for me, too, if I explain our responsibility to help to the child and that the reason we ask is that we want to ensure their well-being” (#3). Although most of the participants found their limited ability to control events in the children’s lives stressful, reminding themselves of their mission helped. “I can’t make things perfect, but to manage to see that you still can make a difference and find some mastery in that helps” (#5). Interestingly, although all participants experienced emotional strain when listening to the children’s stories of abuse, a few participants maintained that being in a position to help made it easier to listen. “It can be difficult to hear about violence. I can’t watch violence in movies, but it’s easier to listen to people telling me about it because then you can contribute, hopefully, and be more than a passive witness to violence as entertainment or news” (#10).

Many participants expressed pride in their work. One participant described her CPS’ mentality by stating that “I find that there’s a pride in what we do and in our profession” (#3). Other participants expressed their pride differently. “Unfortunately, it’s not that often, but it feels meaningful when you can do good and give the child the help [he/she] needs. Then, you think you’ve accomplished something” (#6). “For me, it’s thinking I can make a difference in that child’s life” (#4). Even when the participants became unpopular due to their unwelcomed interference, their self-respect and pride in their work helped them to endure. “I think that if I can commit to what I say, and we have a good rationale that’s in the child’s best interest, and it’s a well-founded argument, they can just go on disliking me. I can stand by my choices regardless, because I’ve done the right thing. And then, it’s fine” (#6). Having their work and intentions align with their core values enhanced the participants’ self-respect and helped them to manage the hardships and challenges associated with their work.

## Discussion

In our study, the participants shared what they perceived to be facilitators for handling challenges and increasing their proficiency when exploring abuse in child interviews. All participants agreed that routines and assessment tools could aid in overcoming inner resistance and in safeguarding decisions, which thus eases the pressure on the child, although some of them worried that routines could diminish professional responsibility and clinical judgement. Discussing their interpretations and decisions with other professionals relieved the participants’ doubts, and they became less fearful of making mistakes. Additionally, by offering comfort, understanding and encouragement, the participants’ colleagues and leaders were deemed to be important in facilitating the participants’ regulation of difficult emotions to promote their self-worth. All participants agreed that practice was crucial to reducing emotional strain and increasing their competence with respect to exploring abuse. Furthermore, they also agreed that exploration required courage, willpower, repeated reflective practice, and the finding of one’s individual work style. Knowing that they did meaningful work that aligned with their core values allowed the participants to tolerate emotionally taxing situations while maintaining their self-respect and believing that they could make a difference.

Our findings indicate that professionals’ facilitators for addressing abuse in child interviews are two-dimensional; some facilitators alleviate the participants’ emotional strain and doubt, while other facilitators promote the participants’ job satisfaction. To help professionals manage difficult emotions and doubt we should offer them emotional support as well as frequent case discussions and joint responsibility for complex decisions and appraisals. Meanwhile in order to increase professionals’ job satisfaction we should provide them with practice time and tailored feedback on their performance as well as promote an awareness of their personal values and put focus on the humanitarian goals of this work.

### Joining children in the minefield: is it all about motivation?

**Fig. 1:**
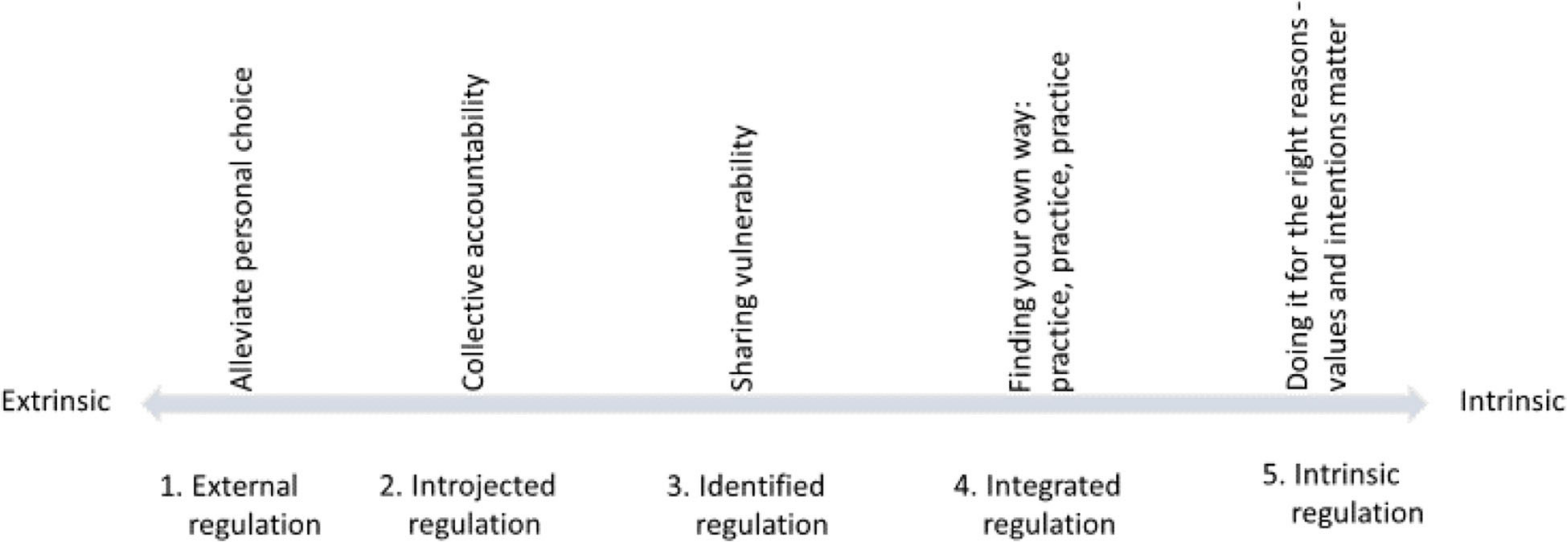
Themes sorted by motivational origin. Elaborated from Ryan & Deci [29]

Given that the facilitators for performing emotionally difficult work are closely related to motivators, we discuss our findings with motivation theory. Ryan and Deci’s self-determination continuum [29] can explain the experienced participants’ identified facilitators when handling the challenges associated with addressing child abuse, including negative emotions and self-doubt. Our themes fit within their model while detailing the different degrees of extrinsic motivation into various regulatory styles. As depicted in Fig. 1, behavioral regulation and the perceived locus of causality are allocated on a continuum from extrinsic to intrinsic motivation. Our theme, alleviating personal choice, corresponds with externally regulated behavior either by compliance or by response to external rewards and punishments. With respect to introjected regulation, behaviors serve to avoid guilt and fear or to enhance pride and uphold contingent self-esteem, both of which concur with our theme of collective accountability. Our theme, sharing vulnerability, equates to identified regulation given that reflecting goals and actions are valued as individually important. Integrated regulation occurs when developing a professional identity as actions and goals become more congruent and begin to synthesize with the self, although they may still be motivated by extrinsic reasons. Finally, intrinsic regulation occurs when actions and goals are inherently gratifying, as in our theme, doing it for the right reasons.

These dissimilar types of motivation are located on a continuum of relative autonomy or self-determination [30]. As increasing levels of autonomy or internalized motivation amplify work engagement and stress-tolerance [31], they may also improve both achievement effort and performance [32]. Furthermore, relatedness and competence facilitate the internalization of goals and behaviors [29], which explains how feeling connected to and cared for by colleagues and how experiencing efficacious performance increased our participants’ mastery of challenges.

### To promote autonomous professionals

Given the benefits of autonomous behavioral regulation, such as effectiveness, persistence, well-being and group cohesion, Ryan and Deci [29] suggest engineering the social environment to facilitate the integration of extrinsic motivation. Such autonomy-supportive contexts must relinquish undue pressure towards acting or thinking in a certain way and instead promote freedom of choice and volition while also guiding professionals to find meaning that they can synthesize with their personal values and goals. Focusing on interpersonal involvement and emotional support in the work environment can enhance professionals’ relatedness. Providing structure in the work-place for goal-directed endeavors, including positive feed-back regarding performance, can increase perceived competence and intensify intrinsic motivation.

Initially, professionals may strive towards preventing discomfort and achieving self-control, but because this leads to only limited self-awareness and self-development, we anticipate that practice and increased emotion regulation skills will shift their focus towards promotion and growth.

Schwartz [33] argues that any work that involves human interaction requires practical wisdom. Practical wisdom entails goodness in conduct and action, both in the form of the moral will to do good and in the ability to discern the right thing to do in any situation. Consequently, practical wisdom can never be attained through external regulation, such as rules, guidelines, or incentives. In contrast, Schwartz contends that extreme dependence on guidelines prevents professionals from developing moral skills and that unwarranted dependence on incentives weakens their moral will. Moreover, because rules and incentives demoralize professionals and erode practical wisdom, it is crucial to encourage professionals to handle their challenges without relying on rules and incentives and to guide them towards intrinsic behavioral regulation.

### Reflexivity

During the data gathering process, the participants may have been reluctant to reveal their insecurities if they perceived the interviewer as an expert on child abuse due to the interviewer’s cited workplace. Accordingly, emphasis was placed on promoting a safe atmosphere and posing open and nondirective questions. The interviewer’s experience providing competence development in CPS and CAMHS may have influenced the probing and directionality of the interviews. Similarly, the fact that the interviewer knew the system and was open to critical viewpoints and opinions may also have influenced the way that the participants responded. Interestingly, many participants said that the interview had expanded their reflections and insights regarding themselves and their work.

When analyzing the data, we strived towards a reflexive awareness of our preconceptions, and we continuously discussed how these reflections might affect our interpretation and condensation into themes. The authors have diverse experiences and fields of expertise within the discipline of psychology, which thus added credibility to our findings. However, researchers from different disciplines, such as sociology or anthropology, may have interpreted the data differently according to their educational background.

### Scope and limitations

We attempted to maximize the range and variety of the participants’ lived experiences in exploring abuse by recruiting participants who varied in age and work experience, were from different geographical locations and worked in organizations of various sizes. Varying the gender distribution, however, was difficult. Our sample include only two male CPS workers and one male CAMHS worker. This reflects the scarcity of men in the workplaces that we contacted and the general gender distribution in these services [34]. As a preliminary analysis revealed no apparent gender differences, we did not expand our recruitment to balance the gender distribution.

## Conclusions

Several important points from these findings should guide future interventions that serve to improve professionals’ skills when handling the challenges associated with exploring child abuse. We recommend promoting the autonomy of professionals by emphasizing the meaningfulness of their work that is compatible with their personal values. Furthermore, the findings indicate that developing personal competence depends on encouraging individual choice and volition while decreasing the demands towards following rules and guidelines. To facilitate the relatedness and the emotion regulation of professionals, our results suggest endorsing shared vulnerability with colleagues and promoting an organizational culture that supports openness and allows practitioners to discuss their emotions when addressing difficult and complex issues. Finally, we recommend competence development in the area of goal-directed reflective practice combined with positive feedback on performance.

## Additional file

Additional file 1: Interview guide. How professionals experience addressing child abuse. Interview guide translated from Norwegian to English. (DOCX 17 kb)

## Abbreviations

CAMHS: Child and Adolescent Mental Health Services
CPS: Child Protective Services
ID: Interpretive Description
