# Supplementary material for: Entering an emotional minefield: professionals’ experiences with facilitators to address abuse in child interviews
Source: BMC Health Serv Res. 2019 May 10;19:302. doi: 10.1186/s12913-019-4128-8 (PMC6511197; doi:10.1186/s12913-019-4128-8)
Supplement: Supplementary file 1 — Interview guide. How professionals experience addressing child abuse. Interview guide translated from Norwegian to English. (DOCX 17 kb) [file 12913_2019_4128_MOESM1_ESM.docx]

**How professionals experience addressing child abuse**

**Interview guide translated from Norwegian to English**

1. Will you please tell me about your experiences asking children and youth if they have been exposed to various types of child abuse?
   1. Can you describe in detail a situation where you felt it was okay to ask?
   2. Can you describe in detail a situation where you felt it was challenging to ask?
   3. What do you think was the reasons for this?
2. Do you have experiences where you either have revealed a child’s exposure to child abuse or cases where you have had a strong suspicion of child abuse?
   1. Will you tell me more about your experience related to this/those cases?
   2. What did you learn from this/those experiences?
3. What, in your opinion, is influential when asking children and youth about adverse experiences?
   1. If relevant, what do you think may have contributed to making you insecure in the situation?
   2. How do you think personal experiences contribute? If you find them influential, would you elaborate on what type of experiences matter?
   3. Could you say some more about how this is interrelated?
4. How do you experience characteristics of your work environment affect how challenging you find it to ask?
   1. How do you feel your relationships with your colleagues influence your asking?
   2. The manner of leadership on your work place, how do you feel that influences how easy or challenging it is to ask?
   3. How do you experience routines and guidelines affect asking the children?
   4. How do you feel the culture at your work place influences you? Are there any unwritten rules that you think affect if it is easy to ask or not?
   5. Are there other factors in the system you work in that you believe can influence how you feel about asking children about adverse events? Do you think the cooperation with other organizations somehow influence how easy it is to ask?
5. How are your experiences of taking care of the child while inquiring about child abuse?
6. Will you please describe as much as possible of things you feel influences your experience of how easy or difficult it is to ask children whether they have been exposed to adverse experiences?
7. What advice would you give to other professionals for them to feel less challenged asking children.
8. What advice Would you give to leaders to make it easier for professionals to ask to children about negative experiences?
9. What advice would you offer to agents providing competence development regarding exploration of child abuse in children and youth? What do you think would make a difference?
